# Indents

**Published:** 1923-07

**Authors:** 


### INDENTS

**☞Brief Articles of Technical or Scientific Value will receive notice and comment. Contributions invited.**

---

INDUSTRIAL DENTISTRY. It is true that much more care is being given to the treatment of dental disease in this country than was the case in former times. The various school clinics are doing great service, and adult clinics, designed to carry on the good work, are springing up in certain centers.

Some of the great industrial firms have also realized the importance of making provision for the dental treatment of their employes. This is a branch of preventive dentistry that is of the first importance, and is capable of enormous development. The task of educating the great mass of the industrial classes, as individuals, in the value of freedom from dental disease, both to the individual and to the community, is a task before the magnitude of which the strongest and most enthusiastic might quail. Individual effort is excellent, but it is not enough. Combined and systematic action is required, in order to instil these principles into the nation as a whole. In this great work the cordial co-operation of the employers of labor is urgently required, if the campaign is to be a success.

The school clinics are fighting the preliminary battle, and they are winning. If all the great industrial firms will provide schemes whereby their employes can obtain regular dental treatment and instruction in dental hygiene, it will be a great step forward towards health and prosperity. —*The Dental Surgeon.*

